# Best Practice Standards in Animal-Assisted Interventions: How the *LEAD* Risk Assessment Tool Can Help

**DOI:** 10.3390/ani10060974

**Published:** 2020-06-03

**Authors:** Victoria L. Brelsford, Mirena Dimolareva, Nancy R. Gee, Kerstin Meints

**Affiliations:** 1 School of Psychology, University of Lincoln, Brayford Wharf East, Lincoln, Lincolnshire LN5 7AY, UK; vbrelsford@lincoln.ac.uk; 2 Division of Psychology, Bishop Grosseteste University, Longdales Road, Lincoln, Lincolnshire LN1 3DY, UK; mirena.dimolareva@bishopg.ac.uk; 3 Center for Human-Animal Interaction, School of Medicine, Virginia Commonwealth University, Richmond, VA 23298-0710, USA; nancy.gee@VCUHealth.org

**Keywords:** animal-assisted interventions, dog-assisted interventions, safety standards, risk assessment tool, risk assessment, risk, best practice recommendations

## Abstract

**Simple Summary:**

Animal-assisted interventions (AAI) in educational and other settings have steadily increased over the last fifty years. While scientific research on the effects of AAI is still growing, the application in the field has overtaken the science and seen a steep rise in many countries and settings in recent years. Surprisingly, while different organisations provide a range of guidelines, no unified, standardised guidelines exist, nor do easy-to-use risk assessment tools for AAI providers and users exist. With differences in practical application and different policies used by AAI providers worldwide, this means that in practice AAI takes place in an unregulated manner without a gold standard of best practice. To ensure safe AAI worldwide, we provide urgently needed unified guidelines on best practice in relation to risk assessment, safeguarding and animal welfare priorities. We also provide the first comprehensive risk assessment and animal welfare tools to achieve consistent welfare and safety standards for best practice across educational and other settings around the world.

**Abstract:**

Animal-assisted interventions (AAI) in educational and other settings have steadily increased over the last fifty years and a steep rise in AAI has been observed in many countries and settings in recent years. Surprisingly, while different providers and organisations provide a range of guidelines, no unified, standardised guidelines or risk assessment tools for AAI exist. This means that in practice AAI takes place in an unregulated manner and without a gold standard of best practice. In addition, knowledge of which interventions are effective is still scarce and the mechanisms of successful interventions are not yet fully understood. This is partly due to AAI being a relatively new research field and standards of research and practice have often lacked rigour in the past. Furthermore, knowledge and experience of providers undertaking interventions varies greatly as there is no standardised training either. We address the striking lack of standardised guidelines and procedures. In all AAI, high importance should be placed on safety and welfare of all involved. Children and other AAI participants, staff and animals should be given equal consideration when assessing risks and welfare needs. To ensure safe AAI worldwide, we provide urgently needed guidelines on best practice in relation to risk assessment, safeguarding and animal welfare priorities. The guidelines were developed for a large-scale longitudinal, randomised controlled trial AAI project and are relevant to AAIs within educational and other settings. We also provide the first set of comprehensive risk assessment and animal welfare tools to achieve consistent welfare and safety standards for best practice across educational and other settings around the world.

## 1. Introduction

Public and scientific interest in the benefits of human–animal interactions (HAI) and animal-assisted interventions (AAI) has been steadily increasing over the last fifty years [1,2,3,4]. Consequently, the inclusion of animals within the educational environment has become increasingly popular [4].

However, despite the surge in AAI, no unified, standardised guidelines for animal-assisted interventions exist, nor are there universal, ethical guidelines relating to human–animal interactions [5], or accrediting or regulatory bodies. Instead, different providers and organisations worldwide provide a range of guidelines and voluntary codes of practice (e.g., Pet Partners, USA; Society for Companion Animal Studies (SCAS), UK; Animal-Assisted Interventions International (AAII)). Both SCAS and AAII have renewed their guidelines in 2019 [6,7]; see also the International Association of Human-Animal Interaction Organizations (IAHAIO) White Paper 2018 [8]. Serpell et al. [9] provide a recent representative survey of current standards and differing practices in the USA. It is noteworthy that in the US, and around the world, many organisations have set some guidelines and policies as to how they prefer to work, but guidance and actual practices differ. This means that, thus far, in settings around the world, AAI takes place in an unregulated manner and without an accepted gold standard of best practice. In addition, there is also a need to standardise training for AAI providers as considerable variation prevails in how knowledgeable providers and their AAI teams are. In the following, we address the striking lack of a risk assessment tool and of standardised guidelines and procedures and contribute to knowledge improvement and awareness in stakeholders and all involved in AAI.

Prior to implementing animal-assisted education (AAE) or to applying AAI in an educational or any other environment, it is important that educators and practitioners understand three essential points.

Firstly, whilst some research shows that animals have been found to enrich educational practices and may support specific tasks such as reading [10], can aid social functioning [11] and can provide social support for children with insecure/disorganised attachment [12,13], it must be noted that there is still a need for rigorous research evidence to provide a robust knowledge base on effective interventions in relation to both typically developing children and children with special needs (for overviews see [14,15,16]. This urges caution concerning, for example, the inclusion of animals in educational settings and highlights the role of senior leaders to design, prepare and monitor their investigations carefully.

AAE or, more generally, AAI, must not be considered a ‘one-size-fits-all’ approach; instead, practitioners should be knowledgeable about existing research and be aware of which practice has previously produced effective outcomes in comparable settings (as demonstrated by robust research findings) and seek appropriate professional guidance before embarking on AAI with children, students, older learners or other participants. This will ensure that the chosen intervention is appropriate to the needs of the individuals concerned and has the potential to produce beneficial results.

Secondly, it is important that educators, therapeutic practitioners and researchers exercise ethical and conscientious decision-making when considering applying AAI sessions within educational or other settings and in the process also eliminate unnecessary contact and/or stress for animals. Consideration must be given to the welfare needs of all involved, including the animals and these should not be compromised in favour of the child, young person or other AAI recipient.

Thirdly, practitioners, educators and others advocating the interaction of animals and humans within educational or other settings should be mindful of the potential risks to both humans and animals. It is crucial that settings undertake hazard identification and implement practical control measures in advance of carrying out AAI or AAE, not least to protect the welfare of animals. This can be achieved through a combination of risk assessment, child/participant safeguarding practices and the implementation of animal welfare protocols.

This article will propose such guidelines and risk assessment practices. We will discuss in detail all areas of safety for animals, children and other participants, as well as staff involved during AAI in educational and other settings. The **L**incoln **E**ducation **A**ssistance with **D**ogs (**LEAD**) risk assessments with safety and welfare tools and guidelines (Tables S1 and S2) and the dog welfare plan (Table S3) were developed by the LEAD team from October 2015 to May 2016. The risk assessment tools were successfully trialled and implemented in the context of a longitudinal randomised controlled trial AAI project, running from 2015–2018 [17]; also see LEAD webpages [18] for more detail on the project: http://lead.blogs.lincoln.ac.uk (publications from the project in progress). Please note that the results of the different project arms are not the point of discussion here and will be reported elsewhere.

The LEAD tools are appropriate for the application of animal-assisted interventions (AAI) overall, animal-assisted education (AAE), but also animal-assisted therapy (AAT) and animal-assisted activities (AAA) (for an overview of types of animal-assisted interventions, see [2]).

Indeed, all interventions with animals, and particularly dogs, will benefit from applying these comprehensive and rigorous risk assessment tools.

## 2. Guidelines for HAI and AAI: The LEAD Risk Assessment Toolkit

The LEAD Risk Assessment Toolkit (Tables S1–S3) is recommended as a comprehensive tool and proactive measure towards achieving high standards of safe and ethical practice within the field of HAI and AAI. The toolkit consists of a school risk assessment tool for dog-assisted interventions, a general settings risk assessment tool and a dog care plan. See Tables S1 and S2 for risk tools and see Table S3 for the dog care plan which is to be used with each of the risk tools. This toolkit allows for a consistent application of risk assessment in relation to managing AAI sessions in various settings, for example by educators, therapeutic practitioners and researchers. It also offers room for flexibility in that it is mindful that educational, therapeutic and other establishments will have their own policies and procedures that must be obeyed. Allowing settings to incorporate their requirements into the tool creates a bespoke risk assessment, suitable for any educational or other setting and for children and learners of all ages and abilities, from nurseries to mainstream schools and special educational needs schools.

### 2.1. Safeguarding and Protection of Children, Young People and Other Participants: Human Contact

Safeguarding children and young people means protecting them from abuse and maltreatment. Safeguarding also includes to keep them safe from harm to their health or their development, letting them grow up with effective care, and enabling them to have the best outcomes for the future [19]). In the UK, local authorities have the overarching responsibility for safeguarding and promoting the welfare of all children and young people in their area. Within this framework, and under regulations made in sections 94 (1) and (2) of the UK’s Education and Skills Act 2008 [20], all educational establishments have a statutory duty to ensure that safeguarding arrangements are in place. Systems for implementing the safeguarding and promotion of welfare of children and young people will vary between establishments; nevertheless, all must have safeguarding policies in place and staff should be made aware of them as they have a responsibility to keep children and young people safe.

For schools, it is best practice to check that all educators, therapeutic practitioners, researchers and animal/dog handlers who are in close contact with pupils are eligible to work with children and young people prior to interventions beginning. Next to obtaining relevant references, a formal way of checking if a person can be allowed to work with children and young people can be to ask for a “police records check” which is provided in a variety of countries. Please note that different countries have different rules as to whether schools or employers are allowed to ask for police records and for which type of record. In the UK, the “Disclosure and Barring Service” (DBS) certificates are of four types: a basic check shows unspent convictions and conditional cautions; a standard check shows spent and unspent convictions, cautions, reprimands and final warnings; an enhanced check shows the same as a standard check, plus information held by the local police considered relevant to the role and a further enhanced check includes barred lists [21]). The DBS carries detailed information on who can be asked to undergo a check and for whom this is not necessary or allowed. The LEAD risk assessment toolkit encourages best practice by advising that in all settings when researchers, educators, practitioners and dog handlers come into direct contact with children/young people or other specific or vulnerable populations, they check if they require the relevant DBS checks prior to intervention commencing.

To further safeguard all participants’ interests, valid consent for participants should be obtained (as outlined for example by BPS Code of Conduct [22], see Chapters 4 and 10). For vulnerable populations, e.g., children under 16 and others lacking the capacity to consent by themselves, caregiver consent will be sought. In addition, participants’ assent to taking part will be obtained prior to any AAI and shall be monitored throughout. AAI should be ceased if any signs of wanting to stop are detected. Other participants will actively consent. All participants can withdraw from the AAI at any time without having to give a reason.

In addition, children will not need to be left alone with dog handlers, but should be supervised by another adult, for example a teacher or teaching assistant, during an animal intervention or animal activity. Ideally, AAI sees the dog handler in charge of the animal and his/her welfare, and an educational or therapeutic practitioner is responsible for the child or other participant and ensuring that the aims of the session are met. It would therefore not be recommended that dog handlers (who are typically not trained as teachers or educators) would be left in sole charge of children or young people during sessions. Instead, they should be free to monitor their dog for the whole session duration. However, a notable exception here consists, for example, of professional counselling psychologists or other therapists who employ their own therapy dog when seeing clients. While such cases still benefit from using the risk assessment toolkit, the nature of the treatment would preclude the presence of another adult.

Overall, researchers, dog handlers and practitioners applying AAI in educational settings must always also adhere to setting policy and participant safeguarding measures should be aligned with the procedures of any interventions implemented.

### 2.2. Safeguarding and Protection of Children, Young People and Other Participants: Animal Contact

All animals should always be treated with respect and dignity (Animal Welfare Act, 2006, [23]) (see also the chapter below). The LEAD risk assessment toolkit focuses on interventions with dogs and advises that all dogs involved in educational or any other interventions be treated appropriately.

Before AAI occurs, all dogs should be assessed by independent assessors with a suitable knowledge of dog behaviour and welfare for their suitability to work with children and young adults or other participants. This is important to protect both the human and the dog’s welfare needs, especially as school environments are often noisy and novel to the animals, and some could become startled and fearful, which should be avoided. Other dogs may become overexcited or boisterous, which could, in turn, cause problems for the researcher, educator or practitioner with maintaining control over the situation and maintaining a controlled environment. This may also heighten the risk of accidental injury and/or enhance the potential for a child or young person to become fearful if they were somewhat uncertain of being around a dog in the first place. Thus, it is crucial that dogs are assessed and judged as healthy and suitable to work with children, young people or other participants as and when this is relevant.

Where AAI is obtained through private and charitable organisations, it is advised that these organisations will have assessed their animals as suitable for interactions within the chosen settings; however, it would be prudent for any educators, practitioners or researchers wishing to engage in such interventions to check this in advance. All in all, we advise that best practice for researchers on AAI projects should involve an objective, external animal behaviourist or a veterinarian with appropriate training in animal behaviour to carry out assessments in a similar setting prior to the interactions to ensure the suitability of animals to be included in projects involving AAIs. Ensuring a current assessment of the dog’s behaviour is important, as health or behavioural triggers may have changed since the initial assessment as a therapy animal, or the environment where the assessment took place may not closely mirror the type of environment the animal will work in. We therefore recommend training of dog handlers and regularly updated assessment of therapy animals by qualified assessors.

Dog handlers should be knowledgeable about their companion animal, should be trained in and able to read their body language and stress signals [24,25], always manage their dog’s behaviour well, and safeguard the welfare of their pet partner at all times. As well as reducing the risk of falls, bites and scratches to the participant through a process of continual reflection and assessment by the handler, it is crucial that handlers act as role models for children and others in promoting compassionate behaviour. Modelling of contact behaviours, instructional guidance and discussions around the animals themselves can be used to achieve this.

In addition, it is essential that children, young people and all other AAI participants are made aware of and understand the reason for ground rules such as not rushing up and crowding the dogs, or hugging or kissing them, but instead showing respect and consideration for the animal’s situation and keeping calm when animals are present. For some children or other participants with special needs who may need support to show restraint or follow instructions, it is important that a trained member of staff assists with mediating their responses. Ground rules for all AAI should be obeyed and set the tone for future interactions—high standards of compassionate behaviour towards animals should be demonstrated and maintained.

To enhance understanding of this and to reduce the risk of nervousness and adverse reactions to dogs, children, young people and all participating in interventions with animals should be given appropriate safety and welfare training in advance of interventions. For dog-assisted interventions, it is crucial that participants are given instruction on understanding stress signalling in dog behaviours [24] as well as information on safe behaviour around dogs [26].

In the unlikely event that scratches and bites do occur, these should be carefully attended to and a first aider be alerted to assess the injury and, if necessary, professional medical attention should be sought. All such incidents should be recorded in accordance with school/setting procedures and logged in accident/incident books as appropriate. Dog bites should also be reported to appropriate health agencies if and as required by local or national governments.

Interactions should be halted immediately where any behaviour from the child/participant or the animal calls into question the safety of the other. It is also vital that all dogs and dog-handler teams have valid and appropriate insurance. The LEAD risk assessment toolkit incorporates all these factors within its assessment process.

Finally, the location for the AAI should be agreed in advance and arrangements made for the appropriate care of the dog, given its size and temperament. This also applies to decisions such as how the animals enter and exit the setting, and an initial familiarisation visit. For instance, bringing a dog through a busy school entrance creates an opportunity for unmanaged interactions and could create discomfort, distress and risks for children or staff with allergies or phobias as well as stress for the dog and should be avoided where possible. School/setting personnel can aid in reducing this unnecessary interaction either by agreeing the use of alternative entrances or advising on suitable times for animals to enter and leave the school. Ideally, a separate room is to be used that is quiet, well-lit and ventilated and can be cleaned easily. Dogs should not be allowed to roam around the setting environment unrestricted and should always be accompanied by a handler, regardless of the size or type of animal.

Concerning hygiene and the reduction of risk from infection and illness, all school and childcare settings in the UK are required to follow guidance published by the Health Protection Agency in relation to infection control. The information is published with assistance from the Royal College of Paediatrics and Child Health [27] and Public Health England [28] and aims to prevent the spread of infections by ensuring routine immunisation, high standards of personal hygiene and practice, and maintaining a clean environment. The LEAD risk assessment toolkit makes clear, that staff in each setting must be aware of their own infection control policies and follow them as appropriate. These can be added to the LEAD risk assessment to create robust but bespoke assessments in-line with individual school policy. Hand-washing is seen as the most important and cost-effective practice to reduce the risk of cross-transmission of infections [29]. In practical terms, most AAI sessions in educational or other settings will not be carried out in a room which has a sink and running water readily accessible. Hand-sanitiser and anti-bacterial wipes should always be made available and should be used at the end of each session where an AAI participant has been in contact with an animal. Hand-sanitiser and anti-bacterial wipes do not replace the process of hand-washing, but should be a defence against germs until participants can wash their hands properly with soap and water. It is good practice for all participants to wash their hands at the end of intervention sessions before starting other activities—this is especially important before snack and mealtimes. Researchers and staff will also need to adhere to good hand-washing practices. Activities such as handling animals’ bedding, water bowls and toys are all points of contact with the potential to spread germs. As well as setting a good example, it sets a standard for good practice in relation to limiting cross-infection where zoonotic diseases are concerned.

Good hygiene practices start with common sense. For example, children or other participants with significant cuts or abrasions on areas of the body which are likely to come into direct contact with an animal such as exposed hands, arms or legs, should have these areas covered. Any waste produced during sessions, whether accidental or routine should always be handled and disposed of whilst wearing disposable gloves. Waste should be disposed of regularly by adults and animal litter boxes should not be accessible to children. All contaminated items and surfaces should be properly cleaned and disinfected in accordance with the individual schools’ health and safety protocols. It is important that cross-contamination does not occur, and that cloths and cleaning products are fit for purpose and disposed of in accordance with the setting’s protocols.

As far as allergies and phobias, diseases and parasites are concerned, the LEAD risk assessment toolkit advises that before embarking on the introduction of AAIs, relevant information should be sought and verified in relation to both visiting animals and the children or other participants who the animals will be in direct contact with.

With respect to the dogs, it would be expected that organisations working to provide AAIs would have appropriate checks in place to ensure that animals working for them are clean, vaccinated, wormed and flea treated. Researchers and practitioners should seek confirmation from handlers by viewing veterinary records for each dog assessed and checking that these are up to date.

With respect to the recent raw meat-based diets (RMBD) debate, research results showed bacteria and parasites in RMBDs, including antibiotic-resistant bacteria [30,31]. It is argued that RMBDs “may be a possible source of bacterial infections in pet animals and if transmitted pose a risk for human beings” and if “non-frozen meat is fed, parasitic infections are also possible”. It was concluded that “the presence of antibiotic-resistant bacteria in RMBDs could therefore pose a serious risk to both animal health and public health—not only because infections with these bacteria are difficult to treat, but also because of the potential of it contributing to a more widespread occurrence of such bacteria” [31], all above citation p. 1. While more research on this matter is needed, given the current state of knowledge, a number of UK health trusts and education authorities will not accept visits from therapy animals fed on raw meat diets. Organisations such as Pets as Therapy (PAT) in the UK, or Pet Partners in the USA preclude animals eating RMBDs from participating in their programmes and we recommend following this advice for animal-assisted interventions.

Information on allergies and phobias in the participants should be gained via a brief questionnaire to parents, caregivers or participants themselves to identify those who may suffer an allergic or phobic reaction to animals. Furthermore, practitioners must be mindful of these when conducting AAI sessions. It would be a mistake to assume that an educational or other environment with no animals on site is an allergen-free zone. Research shows that schools are likely to have relatively high concentrations of pet allergens due to second-hand contamination entering the environment naturally through clothing or footfall, especially where pet ownership is high within the local community [32,33]. Pet allergies are caused by contact with proteins found in the dander (skin flakes), saliva and urine of animals and not through pet hair, as is commonly thought, although pet hair may carry these allergens (Asthma and Allergy Foundation of America (AAFA) [34]; NHS, [35]). High standards of personal and environmental hygiene will help with reducing allergens in the immediate environment and avoiding allowing animals to lick those they interact with is recommended within the LEAD risk assessment toolkit. Pupils or other participants with allergies do not necessarily need to be excluded from AAIs, but this depends on the trigger and severity of the allergy they have; the final decision over participation in sessions would be the responsibility of the participant/caregiver to decide. In the rare case that an allergic reaction did occur, medical assistance should be sought to deal with the situation appropriately. The setting procedure should be followed at all times and any incidents should be recorded appropriately by staff dealing with the incident.

In the questionnaire previously mentioned, parents or caregivers need to be asked if children have specific fears or phobias to the type of animal involved in AAI sessions. Exposing a child or young person, or any other participant, to unnecessary stress through a lack of information regarding phobias would be both unethical and dangerous. This does not mean that children or other participants with phobias cannot take part in sessions if they wish, but that the educator, practitioner, researcher and handler are all fully aware of the participant’s needs and sessions are managed appropriately and in a controlled manner conducive to the requirements of the person concerned. Familiarisation sessions prior to intervention are good practice as they help alleviate anxieties in children and others by allowing them to get used to the animals (and vice versa), and also allows practitioners and researchers to gauge any unidentified anxieties in a controlled environment, therefore reducing the risk of problematic interactions once AAI sessions have begun.

### 2.3. Protection of the Animal

Dog handlers themselves are responsible for ensuring that their animal’s physical and psychological wellbeing is protected and not compromised. The Animal Welfare Act 2006 [23], Dog Welfare Act 2006 and the Dog Health and Welfare Act (Scotland) should always be adhered to. Section 9 of the Act places a duty of care on animal owners. However, it is seen as every person’s responsibility to be mindful in all interactions with animals and to behave compassionately towards them.

Ensuring that a dog welfare plan (see Table S3) is included in risk assessment and is implemented and adhered to in practice can help to maintain best welfare practice. The welfare needs of animals must always be met during intervention sessions, whether involved in research or engaged through other sources to support learning. Part of ensuring animal welfare is to keep interactions positive and to react promptly to an animal showing initial signs of stress or wanting to stop any interaction. The length and intensity of sessions should be carefully considered. Unfortunately, regulations relating to this are lacking, possibly due to a lack of consistent and detailed research investigating which durations of interactions would be best for which animal partners, while still showing the best outcomes in participants [36]. There is also a lack of consistency and understanding relating to the differing needs of the wide variety of animals involved in AAIs—future work will need to explore this. The LEAD risk assessment toolkit outlines the need for the duration and intensity of contact between children or other participants and dogs to be minimised to protect them from unnecessary stress and fatigue, with an overall limit of, at most, 2 h per day as a guideline. However, dogs should always be observed carefully and if the animal shows any signs of tiredness or stress or of wanting to withdraw or stop, immediate cessation of any session has to be instigated. Furthermore, as every animal is different and educational environments and interventions differ in terms of the demands placed on the animal, AAIs should be set up by taking these factors into account.

The dog care plan also notes that water has to be available at all times, that a rest area should be provided for the animal (which children or participants are not permitted to invade) and that dogs have their toileting and exercise needs met by being taken outside of the premises as appropriate. In addition, the dog handler should have access to a dog first aid kit in case the dog sustains any injury while in the educational or any other setting. Animals should not have any current illnesses which may cause them discomfort or pain (e.g., arthritis). Dogs must have a yearly physical exam from a qualified veterinarian, test negative for worms and other parasites, and be current on all vaccines. It is good practice to have senior animals checked more frequently because they may develop physical sensitivities with advancing age, such as arthritis, which can be painful and thus stressful to the animal during the AAI sessions. The LEAD risk assessment toolkit incorporates this need for good practice in that dogs involved in AAE or in AAI, for example, for research projects, have a suitable care plan in place to ensure that all individuals understand the specific requirements of the animal involved; see the dog care plan (Table S3).

Given that the practice of AAI is becoming increasingly popular and may increase from a commercial perspective, it is imperative that risk assessments and following best practice are taken into account when engaging the services of any provider of AAI, or where research is being planned which involves such contact. It would be expected that individual providers publish their guidelines as to how long an animal is exposed to AAI during a working day, including the maximum number of days per week that the animal travels and works. If this is not published, practitioners should ask these questions and decide on the choice of provider strictly based on their ethical treatment of the animals.

## 3. Discussion

The LEAD Risk Assessment tool is extensive and includes all areas discussed in this article. The tool is designed to enable educational and other settings to incorporate their own policy, procedure and wider best practice, making it a comprehensive risk assessment tool tailored to each specific setting. Whilst the addition of information is encouraged, it is not recommended that items are removed; all areas within the risk tool are relevant and should be addressed in order to demonstrate that risk is appropriately managed. The LEAD risk tool highlights the importance of hazard identification and the implementation of control measures to ensure that human–animal interactions do not create unnecessary risk or harm to those involved. Emphasis is placed on the need to safeguard and protect both humans and animals, and robust considerations should be made in advance of any sessions taking place within educational settings. A handler should be responsible for the animals at all times and participants of any age should not be left alone with any animals during or after intervention sessions. It is crucial that handlers understand their dogs; if an animal shows any signs of stress, discomfort or tiredness, the handler must take the decision to terminate the session to protect the welfare of the animal involved. Children and other participants should be taught how to be safe around the dogs they meet and must understand and accept the rules of interaction. The animal’s welfare must always be at the forefront of everyone’s mind, and respect for animals promoted at all times.

Adhering to the LEAD risk tool will improve the safety of all involved and optimise procedures within AAI sessions. Ensuring the welfare of the animals involved is not only morally and ethically appropriate but will also result in less undesirable behaviours from the animal and reduce the risk of AAI sessions being terminated early and unsatisfyingly. Following these advised protocols with all involved will have the following effects: it will help children and other participants to experience positive interactions with dogs; it will assure settings and their governance, as well as dog handlers, that thorough risk assessments have been undertaken and interactions proceed in safety; and it will assist the researcher or supervisor in carrying out their studies or interventions in a controlled manner, while also ensuring that animals are treated with respect and care, promoting wider animal welfare within families and the community.

The current publication of the LEAD risk assessment tools and dog care plan will make it accessible for all interested parties. The tools and dog care plan are easy to implement and roll out, as they are freely accessible. They will additionally be promoted at conferences and with interested stakeholders nationally and internationally to ensure the broadest possible benefit to all interested parties.

## 4. Conclusions

To ensure safe AAI worldwide, we have provided urgently needed practical guidelines for best practice in relation to risk assessment, safeguarding and animal welfare priorities. These guidelines are coupled with the easy-to-use LEAD risk assessment tools, including a dog welfare plan. These tools are the first of their kind and are relevant to all AAIs. They will contribute to establishing risk awareness for all involved in AAI and ensure consistent welfare and safety standards for best practice across educational and other settings around the world.

## Supplementary Materials

The following supplementary materials are available online at https://www.mdpi.com/2076-2615/10/6/974/s1, Table S1: Risk Assessment Tool for dog-assisted interventions in Schools and Educational Settings, Table S2: Risk Assessment Tool for dog-assisted interventions in Other Settings; Table S3: Dog Care Plan.
