# Supplementary material for: Best Practice Standards in Animal-Assisted Interventions: How the LEAD Risk Assessment Tool Can Help
Source: Animals (Basel). 2020 Jun 3;10(6):974. doi: 10.3390/ani10060974 (PMC7341237; doi:10.3390/ani10060974)
Supplement: Supplementary file 1 [file animals-10-00974-s001.pdf]

Supplementary Materials: Best Practice Standards in Animal-Assisted Interventions: How the *LEAD* Risk Assessment Tool Can Help

Victoria L. Brelsford, Mirena Dimolareva, Nancy R. Gee and Kerstin Meints

Table S1. Risk Assessment Tool for dog-assisted interventions in Schools and Educational Settings

**Lincoln Education Assistance with Dogs (LEAD)**<br><br>**Risk Assessment Tool**<br>**for dog-assisted interventions**<br>**in Schools and Educational Settings**	UNIVERSITY OF LINCOLN
**This risk assessment is designed to reduce risk and ensure that interventions take place safely within the school or other educational setting.**<br>**Please take the time to read the document carefully, assess in relation to your setting, sign and return to the intervention supervisor or research team.**<br>Please complete sections A, B, C and D below as required and return to the intervention supervisor/research team for further action.	

PART A: ASSESSMENT DETAILS			
**Area/task/activity:** Dog-assisted intervention in schools<br>**Location of activity:** Room in School/Educational Setting			
**School name:**<br>**Address and Contact details:**		**Name of Person(s) undertaking Assessment:**	
		**Signature(s):**	
**Head Teacher (Name):**		**Date of Assessment:**	

**Signature:**		**Planned Review Date:**<br>**(Minimum 12 months)**	
**How communicated to staff:**		**Date communicated to staff:**	

**PART B: HAZARD IDENTIFICATION AND CONTROL MEASURES**				
**Step 1:**<br>**Identify significant hazards**	**Step 2:**<br>**Identify who might be harmed and how**		**Step 3:**<br>**Identify precautionary measures already in place**	**Step 4:**<br>**Identify person/s responsible**
**List of significant hazards** (anything with potential to cause harm)	**Who might be harmed?**	**Type of harm**	**Existing controls**<br>(Actions already taken to control the risk)	**Name:**
All below:	Pupils, staff	All below:	♦ Staff should also refer to any internal school policy, if existing, in relation to animals on school premises when organising an animal visit to school.	♦
Hygiene	Pupils, staff	Infection, illness	♦ School infection control procedures to be always followed;<br>♦ School health and safety procedures to be always followed;<br>♦ Any significant cuts or abrasions on exposed skin of hands and arms should be covered before contact with the dog;<br>♦ Hand sanitizer gel and antibacterial wipes are provided for immediate use before and after contact with the dog;<br>♦ Pupils and adults always wash their hands soon after contact with the dog (or coming into contact with the dog's bedding, water, toys, etc.) and especially before snack/meal times;<br>♦ Health and safety information, including facts about zoonotic diseases can be accessed through the project website at http://lead.blogs.lincoln.ac.uk.	♦
Allergies, diseases, parasites	Pupils, staff	Illness, allergic reaction	♦ Parents/caregivers are asked to identify any pupils known to have allergic reactions to dogs. These pupils may have restricted access to dogs depending on their allergy trigger;<br>♦ In the rare case that an allergic reaction should occur and does not subside, medical assistance will be sought;<br>♦ The dogs will have been regularly taken to a veterinarian and have been recently dewormed and treated for fleas; dogs licking participants should be avoided;	♦

			♦ All waste produced, whether accidental or routine, is handled and disposed of hygienically and contaminated items and surfaces properly washed and disinfected in accordance with schools health and safety procedures.	
Phobias	Pupils, staff	Stress, adverse phobic reaction	♦ Parents/caregivers asked to identify pupils known to have a phobia or fear reaction of dogs;<br>♦ All children will have familiarisation sessions before the interventions begin to ensure confidence and comfort levels of the children involved;<br>♦ Where there are pupils with phobias, dogs are not banned from coming into school, but every effort is made to segregate dogs from those with phobias.	♦
Safeguarding and protection of children: Human behaviour	Pupils	Dog handlers and researchers	♦ All researchers, educators and practitioners and dog handlers will check if they need a safety check carried out through the Disclosure and Barring Service (DBS check) or equivalent and will obtain one if deemed necessary;<br>♦ Valid consent/assent obtained from human participants/caregivers as appropriate<br>♦ Participants can withdraw at any time without having to give reasons; vulnerable groups/assent givers should be monitored for signs of wanting to withdraw and AAI should be stopped if such signs are detected;<br>♦ Children will never be left alone with dog handlers and will always be supervised when in the presence of a dog.	♦
Safeguarding children and adults: Dog behaviour	Pupils, staff	Potential risk of bites, scratches	♦ Checks are carried out by the research team/interventions supervisor prior to the visit to ensure that the dogs are clean, healthy and suitable to work with children present and have valid insurance;<br>♦ Dogs and dog-handler teams have valid and appropriate insurance;<br>♦ Dogs are always closely supervised by their handler;<br>♦ Dogs will not be allowed to wander unrestricted around the school;<br>♦ Pupils are closely supervised by an adult during intervention;<br>♦ Pupils are given safety training with regards to behaviour around dogs prior to interaction with the dog;<br>♦ Pupils will be taught to recognise stress signalling in dog behaviour prior to interaction with a dog as part of safety training;<br>♦ Access to a First Aider and First Aid kits are provided in school;<br>♦ In the unlikely event that any dog scratches or bites may occur, these are carefully washed, and a first aider contacted immediately;<br>♦ Any incidents to be recorded in accordance with school procedures and logged in incident/accident books as appropriate and, if necessary, professional medical attention should be sought.	♦

Protection of dog	Dogs	Stress	♦ The dog handler is responsible for the dog and for ensuring that their dog's physical and psychological wellbeing is protected and not compromised;<br>♦ Dog first aid kits will be provided, and dog handlers are responsible for any first aid administered to dogs;<br>♦ The Dog Welfare Act (2006) and the Dog Health and Welfare Act (Scotland) or equivalents should always be adhered to. These laws apply to all dog owners/keepers, but it is every adults' responsibility to be mindful of this guidance in their interactions with the dog. (see fawc.org.uk/freedoms);<br>♦ Dogs will be monitored for signs of stress by their handler and the researcher/intervention supervisor and removed from the situation should they judge the animal to be stressed or in discomfort;<br>♦ Dogs will be familiarised with the setting, given access to water and an appropriate area for rest, toileting and exercise. Children will not interact with the dog at these times;<br>♦ Dogs will work no longer than 2 h per day in direct contact with children;<br>♦ If at any time during the intervention sessions a dog's welfare is in in danger of being compromised, the session will be stopped immediately;<br>♦ Dogs will have a care plan (see below) in place during their participation in the project<br>♦ A specialist consultant is assigned to the project and can be contacted for advice and guidance on dog welfare, behaviour and training throughout the study if required.	♦

I certify that the risk assessment above fully applies to the area/task/activity under assessment

in:………………………………………………………………………… (Name of school)

Date:
Signed: Name: Risk Assessor.

Do not sign off above if further actions are required (see below Parts C and D for further action).

Parts C and D:
If further action is required or there are further local significant hazards you think should be added, please record these here in *Part C* and transfer any actions required to the *Action Plan in Part D*, then sign off below.
Please return this document and the Action Plan in Part D to the intervention supervisor / research team so that any additional issues can be acknowledged and acted upon asap.

PART C: HAZARD IDENTIFICATION AND CONTROL MEASURES					
**Further Significant hazards**	**Who might be harmed?**	**Type of harm**	**Existing controls** (Actions already taken to control the risk)	**Further action/controls required** (transfer to action plan at Part D below)	**Person responsible**

PART D: ACTION PLAN						
**No.**	**Action required**	**Person(s) responsible to undertake action**	**Priority**	**Projected time scale**	**Notes/comments**	**Date completed**

I certify that the assessment for the task/activity above covers all the significant hazards applicable:

in ……………..…………………………………………………….. (Name of school)

Date:
Signed: Name: Risk Assessor.

Table S2: Risk Assessment Tool for dog-assisted interventions in Other Settings

**Lincoln Education Assistance with Dogs (LEAD)**<br><br>**Risk Assessment Tool**<br>**for dog-assisted interventions**<br>**in Other Settings**	UNIVERSITY OF LINCOLN

This risk assessment is designed to reduce risk and ensure that interventions take place safely within the setting.
Please take the time to read the document carefully, assess in relation to your setting, sign and return to the intervention supervisor or research team.
Please complete sections A, B, C and D below as required and return to the intervention supervisor/ research team for further action.

PART A: ASSESSMENT DETAILS

Area/task/activity: Dog-assisted intervention
Location of activity: Room in Setting

**Setting name:**<br>**Address and Contact details:**		**Name of Person(s) undertaking Assessment:**	
		**Signature(s):**	
**Lead/Head of Setting (Name):**		**Date of Assessment:**	
**Signature:**		**Planned Review Date:**<br>**(Minimum 12 months)**	
**How communicated to staff:**		**Date communicated to staff:**	

PART B: HAZARD IDENTIFICATION AND CONTROL MEASURES				
**Step 1:**<br>**Identify significant hazards**	**Step 2:**<br>**Identify who might be harmed and how**		**Step 3:**<br>**Identify precautionary measures already in place**	**Step 4:**<br>**Identify person/s responsible**
**List of significant hazards** (anything with potential to cause harm)	**Who might be harmed?**	**Type of harm**	**Existing controls**<br>(Actions already taken to control the risk)	**Name:**
All below:	Participants, staff	All below:	♦ Staff should also refer to any internal setting policy, if existing, in relation to animals on premises when organising an animal visit to the setting.	♦
Hygiene	Participants, staff	Infection, illness	♦ Setting infection control procedures to be always followed;<br>♦ Setting health and safety procedures to be always followed;<br>♦ Any significant cuts or abrasions on exposed skin of hands and arms should be covered before contact with the dog;<br>♦ Hand sanitizer gel and antibacterial wipes are provided for immediate use before and after contact with the dog;<br>♦ Participants and adults always wash their hands soon after contact with the dog (or coming into contact with the dog's bedding, water, toys, etc.) and especially before snack/meal times;<br>♦ Health and safety information, including facts about zoonotic diseases can be accessed through the project website at http://lead.blogs.lincoln.ac.uk.	♦
Allergies, diseases, parasites	Participants, staff	Illness, allergic reaction	♦ Participants/caregivers are asked to identify participants known to have allergic reactions to dogs. These participants may have restricted access to dogs depending on the allergy trigger;<br>♦ In the rare case that an allergic reaction should occur and does not subside, medical assistance will be sought;<br>♦ The dogs will have been regularly taken to a veterinarian and have been recently dewormed and treated for fleas; dogs licking participants should be avoided;<br>♦ All waste produced, whether accidental or routine, is handled and disposed of hygienically and contaminated items and surfaces properly washed and disinfected in accordance with settings' health and safety procedures.	♦
Phobias	Participants, staff	Stress, adverse phobic reaction	♦ Participants/caregivers are asked to identify participants known to have a phobia of dogs;<br>♦ All participants will have familiarisation sessions before the interventions begin to ensure confidence and comfort levels of the participants involved;	♦

			♦ Where there are participants with phobias, dogs are not banned from coming into setting, but every effort is made to segregate dogs from those with phobias.	
Safeguarding and protection of participants: Human behaviour	Participants	Dog handlers and researchers	♦ All researchers, educators and practitioners and dog handlers will check if they need a safety check carried out through the Disclosure and Barring Service (DBS check) or equivalent and will obtain one if deemed necessary;<br>♦ Valid consent/assent obtained from human participants/caregivers as appropriate;<br>♦ Participants can withdraw at any time without having to give reasons; vulnerable groups/assent givers should be monitored for signs of wanting to withdraw and AAI should be stopped if such signs are detected;<br>♦ Participants will never be left alone with dog handlers and will always be supervised when in the presence of a dog.	♦
Safeguarding participants:<br>Dog behaviour	Participants, staff	Potential risk of bites, scratches	♦ Checks are carried out by the research team/interventions supervisor prior to the visit to ensure that the dogs are clean, healthy and suitable to work with participants present and have valid insurance;<br>♦ Dogs and dog-handler teams have valid and appropriate insurance;<br>♦ Dogs are closely supervised by their handler at all times;<br>♦ Dogs will not be allowed to wander unrestricted around the setting;<br>♦ Participants are closely supervised by an adult during intervention;<br>♦ Participants are given safety training with regards to behaviour around dogs prior to interaction with the dog;<br>♦ Participants will be taught to recognise stress signalling in dog behaviour prior to interaction with a dog as part of the safety training;<br>♦ Access to a First Aider and First Aid kits are provided in the setting;<br>♦ In the unlikely event that any dog scratches or bites may occur, these are carefully washed, and a first aider contacted immediately;<br>♦ Any incidents to be recorded in accordance with setting procedures and logged in incident/accident books as appropriate and, if necessary, professional medical attention should be sought.	♦
Protection of dog	Dogs	Stress	♦ The dog handler is responsible for the dog and for ensuring that their dog's physical, psychological and emotional wellbeing is protected and not compromised;<br>♦ Dog first aid kits will be provided, and dog handlers are responsible for any first aid administered to dogs;<br>♦ The Dog Welfare Act (2006) and the Dog Health and Welfare Act (Scotland) or equivalents should always be adhered to. These laws apply to all dog owners/keepers, but it is every	♦

			adults' responsibility to be mindful of this guidance in their interactions with the dog. (see fawc.org.uk/freedoms);<br>♦ Dogs will be monitored for signs of stress by their handler and the researcher/intervention supervisor and removed from the situation should they judge the animal to be stressed or in discomfort;<br>♦ Dogs will be familiarised with the setting, given access to water and an appropriate area for rest, toileting and exercise. Participants will not interact with the dog at these times;<br>♦ Dogs will work no longer than 2 h per day in direct contact with participants;<br>♦ If at any time during the intervention sessions a dog's welfare is in in danger of being compromised, the session will be stopped immediately;<br>♦ Dogs will have a care plan (see below) in place during their participation in the project;<br>♦ A specialist consultant is assigned to the project and can be contacted for advice and guidance on dog welfare, behaviour and training throughout the study if required.	

I certify that the risk assessment above fully applies to the area/task/activity under assessment

in ……………………………………………………………………………. (Name of setting)

Date:
Signed: Name: Risk Assessor.

Do not sign off above if further actions are required (see below Parts C and D for further action).

Part C and D:
If further action is required or there are further local significant hazards you think should be added, please record these here in *Part C* and transfer any actions required to the *Action Plan* in *Part D*, then sign off below.
Please return this document and the Action Plan in Part D to the intervention supervisor / research team so that any additional issues can be acknowledged and acted upon asap.

**PART C: HAZARD IDENTIFICATION AND CONTROL MEASURES**					
**Further Significant hazards**	**Who might be harmed?**	**Type of harm**	**Existing controls** (Actions already taken to control the risk)	**Further action/controls required** (transfer to action plan at Part C below)	**Person responsible**

**PART D: ACTION PLAN**						
**No.**	**Action required**	**Person(s) responsible to undertake action**	**Priority**	**Projected time scale**	**Notes/comments**	**Date completed**

I certify that the assessment for the task/activity above covers all the significant hazards applicable:

in………………………………………………………………….. (Name of setting)
Date:
Signed: Name: Risk Assessor.

Table S3: Dog Care Plan

Dog Care Plan

Name of dog:______________________________Age:______________________Gender: ____________Breed: _____________________________________

Insurance statement of handler with signature:

Indicate type of insurance (e.g., public liability insurance with legally binding indemnity):____

for dog (name):______________________________valid (from – to):____

to cover any claims made by a setting in case of an incident during an AAI visit.

Name of handler: ____Signature handler: _______________________________Date:_______

This Care Plan pertains to the welfare of dogs employed during intervention sessions with participants in any setting.
The Care Plan is part of, and should be read in conjunction with, the settings Risk Assessment document.
Areas relating to safety training with participants and teaching safe behaviour with dogs are detailed in the main risk assessment.

**Care and treatment**	**Behaviour management**	**Feeding and watering**	**Toileting**	**Enrichment**	**Exercise**
• Visual health-checks carried out by handler before dog begins work in school/other setting;<br>• Dog familiarised with setting;<br>• Dogs will be monitored throughout sessions to ensure their care, cleanliness and treatment is maintained to a high standard; including child/participant behaviour monitoring to ensure the dog is treated with respect;<br>• Dog handlers will have an animal first aid kit in order to administer emergency first aid in circumstances where this may be required;<br>• Dog not to work more than 2 h per day.	• During the intervention sessions, dog handlers and researchers / supervisors trained in dog distress signalling will carry out constant observations of the child/dog interactions to detect signs of stress in the dogs;<br>• If a dog becomes stressed, the dog will be removed from the situation to protect the welfare of all concerned and allow the dog to feel comfortable in their surroundings.	• Dogs will be fed before arrival at the school;<br>• Dogs will have constant access to water;<br>• Preferably no treats to be given; however, handler to decide on treats / rewards throughout sessions as they see fit;<br>• No treats to be given from hands of participants.	• Designated areas for toileting will have been previously agreed between the school / other setting, the researchers / supervisors and dog handlers in advance;<br>• Dogs will be taken outside at regular intervals as the handler sees fit, or as required by the dog during intervention sessions with the child / other participant.	• Dogs may have a toy in the sessions to enable them to display their natural behaviours;<br>• Dogs will have a bed / blanket in a designated space in the room as a rest area away from human interaction, and they should be given the opportunity to use it as they wish;<br>• Children / participants will not be permitted to approach the dog when the dog is in their resting space.	• Designated areas for exercise will have been previously agreed between the school, the researchers and dog handlers in advance;<br>• When toileting, the dog will be given time to exercise outside in order to enable them to display their natural behaviours and also as a break from direct contact with children.
